# Experimental evolution of protein–protein interaction networks

**DOI:** 10.1042/BJ20130205

**Published:** 2013-07-12

**Authors:** Betül Kaçar, Eric A. Gaucher

**Affiliations:** *NASA Astrobiology Institute, Mountain View, CA 94035, U.S.A.; †School of Biology, Georgia Institute of Technology, Atlanta, GA 30322, U.S.A.; ‡Blue Marble Space Institute of Science, Seattle, WA 98145, U.S.A.; §School of Chemistry and Biochemistry, Georgia Institute of Technology, Atlanta, GA 30322, U.S.A.

**Keywords:** ancestral sequence reconstruction, evolutionary biochemistry of protein network, experimental evolution, network evolution, protein interaction network, AK, adenylate kinase, ASR, ancestral sequence reconstruction, EF-Tu, elongation factor Tu

## Abstract

The modern synthesis of evolutionary theory and genetics has enabled us to discover underlying molecular mechanisms of organismal evolution. We know that in order to maximize an organism's fitness in a particular environment, individual interactions among components of protein and nucleic acid networks need to be optimized by natural selection, or sometimes through random processes, as the organism responds to changes and/or challenges in the environment. Despite the significant role of molecular networks in determining an organism's adaptation to its environment, we still do not know how such inter- and intra-molecular interactions within networks change over time and contribute to an organism's evolvability while maintaining overall network functions. One way to address this challenge is to identify connections between molecular networks and their host organisms, to manipulate these connections, and then attempt to understand how such perturbations influence molecular dynamics of the network and thus influence evolutionary paths and organismal fitness. In the present review, we discuss how integrating evolutionary history with experimental systems that combine tools drawn from molecular evolution, synthetic biology and biochemistry allow us to identify the underlying mechanisms of organismal evolution, particularly from the perspective of protein interaction networks.

## INTRODUCTION

Whether natural or artificial, components in a complex system rarely operate in an isolated manner. In a complex system, components interact with each other through dynamic functional networks [1]. Living organisms are no exception to this observation; crucial cellular functions in a biological system are carried out through intricate biomolecular networks that constantly process vast amounts of internal and external information [2,3]. Like many things in a biological system, molecular networks are inherited and optimized products of evolutionary processes [4]. In fact, many of life's molecular networks have been maintained throughout the evolution of life on Earth for >3.5 billion years [5].

Even though the main structure and function of an essential network may be conserved among divergent species over history, the individual components of the network may exhibit divergent properties [6]. For instance, the overall topology of an interaction network in a thermophilic bacterium can more or less be conserved in a mesophilic homologue despite the disparate adaptive environments of these two species. More specifically, the individual network components in the thermophilic bacterium have to be stable at high temperatures in order for the whole network to carry out its basic function. The mesophilic counterparts are not stable at high temperatures and thus individual components have not had to adapt to extreme environments. Thus functionally divergent properties may be observed at the molecular level despite selection maintaining structurally conserved properties at the network level. The underlying adaptive mechanisms that lead to this dichotomy are a growing interest to the fields of molecular evolution and synthetic biology. Advances in laboratory and computational technologies now allow us to ask, what types of interactions both within and between protein networks influence evolutionary innovations? Under changing environmental conditions, do protein networks conserve their robustness by maintaining a degree of plasticity or promiscuity, or does a network shift its dynamic equilibrium by taking big adaptive steps towards optimality? Do evolutionary responses at the molecular level directly correlate with changes at the organismal level? Furthermore, to what degree can we direct or control a network's function? Comprehensive answers to these challenging questions will reveal links between protein network evolution and organismal fitness. These answers also provide a foundation for our attempts to synthetically design networks and network components that perform desired functions.

In the present review, we discuss how novel approaches in evolutionary and synthetic biology [specifically experimental evolution and ASR (ancestral sequence reconstruction)] can further our understanding of how molecular networks such as protein interaction networks evolve and how understanding the evolution of protein networks can contribute to the analyses and improvement of natural biological systems. We start with a brief review of networks in general and then focus on protein interaction networks and the individual components involved in these networks. We proceed with a discussion on the use of laboratory experimental evolution studies and how these contribute to our understanding of networks. We end with a discussion about how integrating evolutionary history can further our understanding of network evolution.

## BIOLOGICAL NETWORKS

Self-sustaining networks that carry out prebiotic chemistries may have existed before the emergence of cellular life [7]. However, as life evolved and became more complex, so did the biological networks that composed living organisms [8]. One way for biological networks to increase complexity is by recruiting other macromolecules into the existing architecture without diminishing a network's fundamental function. To achieve this, biological networks must exhibit and maintain a certain minimum level of robustness, modularity and plasticity [9–14]. In order to understand the evolution of complex biological networks, therefore not only must we understand how parts of a network co-adapt with the overall network as a whole, but we must also elucidate the evolutionary connections between a host's internal network and the surrounding environment in which the host, and thus the network, both function. The hierarchical behaviour of biological networks poses unique challenges to uncovering these connections in a systematic way [5,15–17]. Although an essential network maintains global information processing to ensure organismal survival, the failure of one node (member of a network) may directly disturb another node or possibly the whole network. Similarly, a change in a hub (main connection point) may influence the network even more substantially than a node, since hubs typically consist of more connections. In other words, in a given biological network not every component is functionally or ‘socially’ equivalent, and each may affect the dynamics of the network, and hence the organism, in different ways.

Owing to the nature of such hierarchical structure of complex systems, the study of how protein interaction networks function requires multiple levels of understanding. First, we must recognize that proteins have independent behaviours; they carry out specific functions irrespective of the network and they must maintain this function while interacting with other proteins in the network. Secondly, structural specificity and stability are important for individual proteins to allow protein–protein interactions. Similar to enzymes, these interactions can be terminated by a single amino acid replacement in the primary sequence that propagates through to the tertiary structure. By contrast, absolute structural rigidity of proteins in the network would prohibit functional adaptation of the network. Thirdly, the topology of the protein interaction network has to be maintained for the network's functionality. Fourthly, each network may behave in singularly novel ways, making general rules difficult to formulate on the basis of the study of individual networks.

A range of approaches may be used to study and describe protein interaction networks. Various mathematical and graph-based models are exploited and provide input to biological studies [18–24]. Several studies followed a comparative approach, wherein the components of the interaction network are compared with the networks in evolutionarily related organisms, and thus enabling a wide-range analysis of the evolutionary organization of protein interaction networks across species [6,25–34]. Among these, various sequence alignment studies have uncovered evolutionarily conserved protein domains in networked systems [32,35–37]. Several computational databases also follow a comparative approach (such as KEGG [38] and BioCyc [39]) and highlight the molecular pathways within protein interaction and metabolic networks [40]. Large sets of empirical data describing the physical properties of protein interaction networks are produced mainly through two hybrid systems, pull-down assays and TAP-MS (tandem affinity purification coupled MS) [41–44]. Lastly, genetic studies are conducted to examine the system-wide phenotypic consequences of a mutation to a protein node as well as changes in environmental selection pressures [41,45–51].

In total, these studies have provided unprecedented means of understanding how biological protein networks evolve. However, there remains a limitation when it comes to making biologically relevant observations. Comparative studies are informative because they provide information about the conservation and lineage-specific aspects of networks, but they are limited because they do not provide molecular mechanisms that describe the functionality of the network. Theoretical studies are a good first-order approximation of complexity, but are typically context-independent and therefore do not capture many of the details that govern specific network evolution. Biochemical studies mainly consider physical interactions of proteins; this reductive approach provides details at the molecular level, but fall short when modelling biological fitness as a function of network properties. Lastly, systems studies ideally provide information about entire systems, but often lack information at the molecular level.

Considering the complexity of factors involved in protein network evolution, an interdisciplinary approach where various methods from biochemistry, and structural and molecular biology are combined with evolutionary and systems biology could potentially bring empirical means to addressing this challenge. The premise is that protein–protein interactions within networks existing today are the product of millions of years of Darwinian evolution at the organismal level. Thus we need to invoke evolutionary history of both the organism and the network components into our model systems in order to make biologically relevant conclusions concerning the functions of complex protein interaction networks.

## HOW DO BIOLOGICAL NETWORKS EVOLVE?

**Figure 1:**
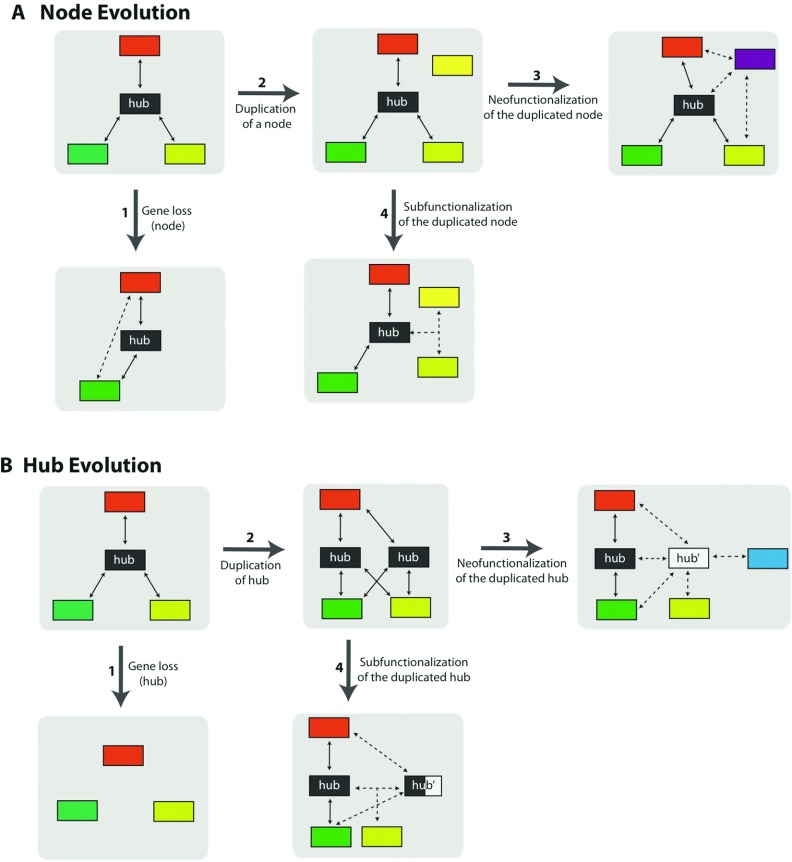
Pathways for network evolution While maintaining the essential function of the network, both hub and nodes can respond to environmental stimuli in a variety of ways. Components of a network, nodes (**A**) and/or a hub (**B**), can undergo various changes during the course of evolution and such changes may directly disturb another component of a network or possibly the whole network. The various examples for how a network can evolve are outlined. Pathway 1: gene deletion. A node whose function and connectivity has subtle effects on the network's phenotype could be lost. If the loss of this node causes a fitness decrease in the system, new interactions between the other members of the network could emerge (**A**). In the case of a hub loss, however, the interactions of the work could be lost, diminishing the whole network's (and potentially the system’s) viability (**B**). Pathway 2: gene duplication. A new compensatory node or a hub could be added to the network through gene duplication. There are various evolutionary trajectories in which this duplicated component can follow. Pathway 3: neofunctionalization. One of the copies of the duplicated gene can accumulate mutations that confer a novel function. Pathway 4: subfunctionalization. A malfunctioning node or a hub could be assisted by the duplicated gene to maintain the network's primary function.

In a constantly evolving system, the individual components of a protein network must adapt to a changing environment while maintaining the network's primary function. How do protein–protein interaction networks maintain such robustness while sustaining their function under fluctuating environmental conditions? There are several ways a protein network can achieve this. For example, a network's robustness could be maintained through gene duplication: a new compensatory node could be added in the network, or alternatively, through gene deletion, a node, whose function and connectivity has subtle effects on the network, could be lost [52] (Figure 1). Additionally, both copies of a duplicated gene could acquire new mutations that are a complimentary subset of the pre-duplication gene's functions [53]. Alternatively, one of the copies of a duplicated gene can be removed from selective pressure due to functional redundancy, and thus under neutral evolution may accumulate mutations that confer a novel function [54,55]. Another way for the network to sustain its robustness could be through retaining a degree of modularity [9,17]. A malfunctioning node could be replaced with another node that provides a selective advantage for the host, through a loss and/or gain of a link (biochemical and/or biophysical reaction) within the network. Lastly, a network can maintain its evolvability by developing more patterns of interactions, and hence can grow in size [56,57].

There are clearly multiple ways in which evolution operates when it comes to maintaining or changing a network's response to a particular environmental pressure. The underlying cellular mechanisms that give rise to network evolution, however, may not be obvious. For instance, if we were to alter a given network in such a way that the directed modification is able to decrease the host organism's fitness, in what adaptive ways would the network/system compensate to increase the host's fitness? One way to better understand the underlying molecular principles contributing to network robustness and evolvability therefore would be to experimentally study how networks (natural or synthetically designed) respond to a given environment, and then to systematically explore the changes as adaptations take place in real time.

The ability to monitor evolution in action through experimental evolution can offer valuable model systems to better understand how molecular networks respond to their environments. Experimental evolution provides a useful platform to study organisms under controlled environments and then monitor their adaptation [58,59]. To systematically explore how the adaptive responses of a given network influence the global properties of an organism, experimentally evolved organisms may be analysed by high-throughput screening techniques. With careful experimental design, not only can we narrow down the focus of evolution as a tinkering effect operating at a network level, but also, through implementing systems biology and biochemical dissection in our analyses, we can understand the system as a whole.

There are several ways to create such a set-up in the laboratory. For instance, based on the notion that highly connected nodes are more important for the network's function than nodes with fewer connections [19,41], a change in the hub of an essential network is most likely to trigger not only a network-level malfunction, but also a subsequent system-level malfunction (Figure 1B). Following this notion, mutating a hub protein of an essential network is most likely to have negative fitness affects. Once such a system is created, a mutant strain hosting the altered network can then be evolved under controlled experimental conditions, and system-level adaptive responses can be subsequently tracked using whole-genome sequencing analyses. In fact, an experimental study monitoring genome-wide responses of regulatory networks in bacteria demonstrated that, when disrupted, hubs have a higher tendency to affect the system-level robustness [46].

The disruption of hub genes can therefore impose a biochemical and topological defect on a bacterial system. This begs the question as to whether, if given a chance to evolve, beneficial mutations would accumulate at the hub in order to re-establish higher fitness. Obviously performing such an experiment is not easy, as deletion of a crucial component of cell machinery is likely to compromise cellular viability. Beyond disrupting network components through insertion/deletion mutations, one alternative experimental approach would be to swap the hub or the node of a network with its evolutionary homologue and then allow the system to adapt under laboratory conditions.

For instance, Shamoo and colleagues have conducted such intriguing experiments with the enzyme AK (adenylate kinase) [60–62]. Representing an important network on the basis of its role in the cellular homoeostatic network, AK influences various metabolic pathways in the cell [63]. In addition, homologues of this enzyme from thermophilic and mesophilic organisms exhibit different dynamic and functional properties [64]. Shamoo's group replaced the thermostable AK with its mesostable homologue in thermophilic bacteria. Such replacement triggered a fitness decrease in the bacterial host. Furthermore, it was shown that when given a chance to evolve in a nutrient-rich environment, the mutational pathways traversed by the thermophilic bacteria hosting a mesostable AK were highly constrained and the only mutations that were observed repeatedly converged to the exact same mutant AK sequence. That study demonstrated that an orthologous gene from a mesophile can replace the function of an endogenous gene in a thermophilic host, albeit with much lower fitness. Furthermore, the study demonstrated that mutational trajectories may be so constrained that the same evolutionary outcome can be obtained repeatedly.

**Figure 2:**
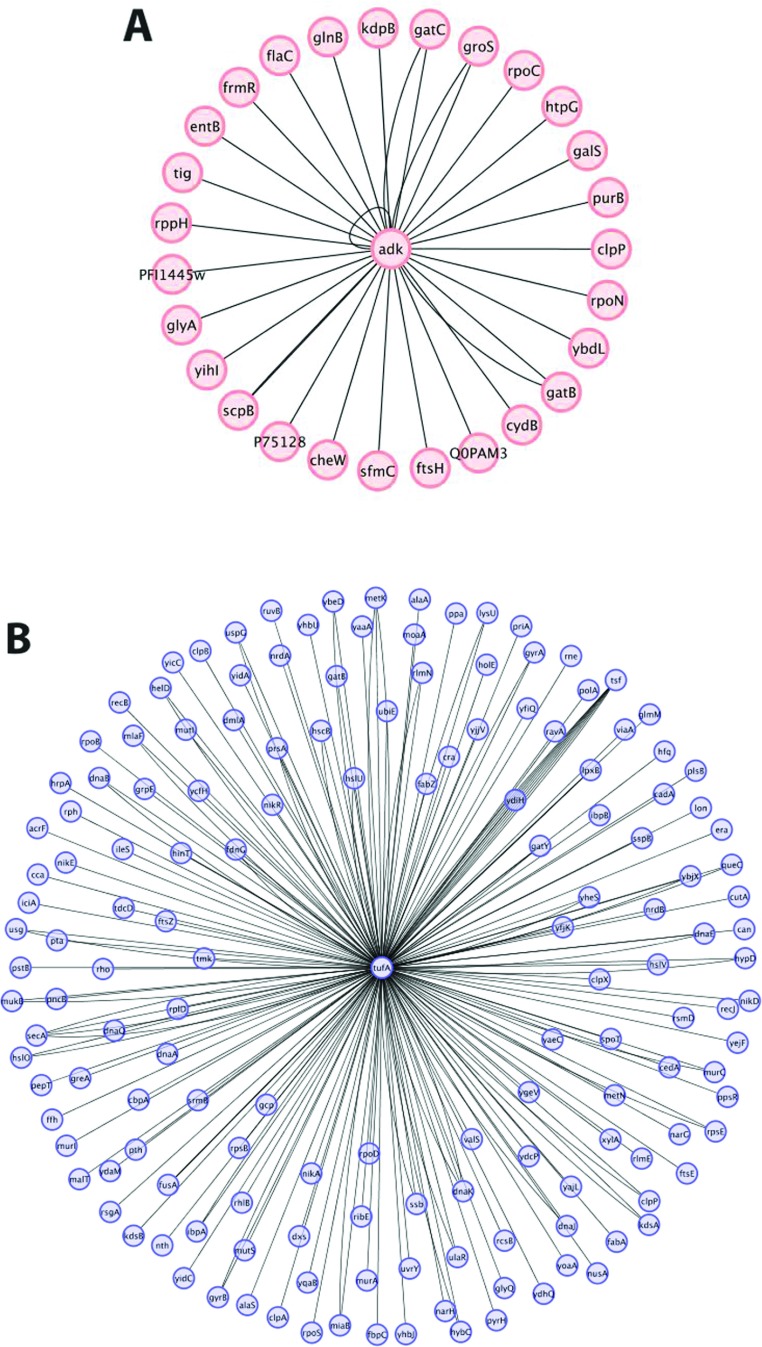
Biological networks The disruption of hub genes can impose a biochemical and topological defect on the biological systems. AK (**A**) and EF-Tu (**B**) are two examples of proteins serving as hubs in essential biological networks. Both network datasets were rendered using Cytoscape 2.8 [85]. (**A**) AK enzyme (shown in the centre as *adk*), serves as a hub through its role in the cellular homoeostatic network [63] and interacts with multiple biological partners. (**B**) EF-Tu protein (shown in the centre as *tufA*), interacts with >100 cellular partners including the ribosome, chaperones, amino acids and tRNAs, and many others. Illustrated are the >50 protein partners that EF-Tu is known (or predicted) to interact with.

Such a result may be expected for multiple reasons. The fact that AK is a hub protein means that it interacts with multiple protein partners (Figure 2A). Thus the ability of the mesostable AK to accumulate mutations that allow it to become thermostable would, in turn, lead to a more stable network. The repetitive nature of the outcome may be due to the fact that the bacterial system is constantly fed and in a closed system, the selection pressure may be so narrow that alternative mutational trajectories cannot be explored. Relaxing the selection pressure in this system may generate alternative mutational paths. Along these lines, Rosenzweig and colleagues showed recently that different experimental selection pressures can lead to different evolutionary outcomes [65].

The above studies provide valuable insights into the evolution of thermostability from the perspectives of protein biochemistry and systems-level biology. However, these studies are limited when it comes to answering our primary question: what evolutionary mechanisms cause essential networks to maintain their robustness? Observing a fitness decrease after replacing an essential cellular component with an evolutionarily related homologue is expected; after all, these homologous enzymes have been evolving within organisms that have themselves been evolving under drastically different selection pressures for millions of years. What if we could track the historical patterns of a network's components through the trajectory that has already taken place? In other words, if we had the ability to go back and resurrect the ancestral states of a network's hub and then replace its modern counterpart with this ancestral component, what would we observe?

Current advances in synthetic biology allow us to attempt precisely this by providing a tool to reprogramme biological networks through integration of evolutionary history [66]. Simply stated, the overall objective here is rewind the molecular tape of a protein's history, reconstruct the molecular history of a given network component through phylogenetic analysis, resurrect the inferred protein sequence of the evolutionary ancestor using laboratory techniques, and then insert it into a modern organism.

**Figure 3:**
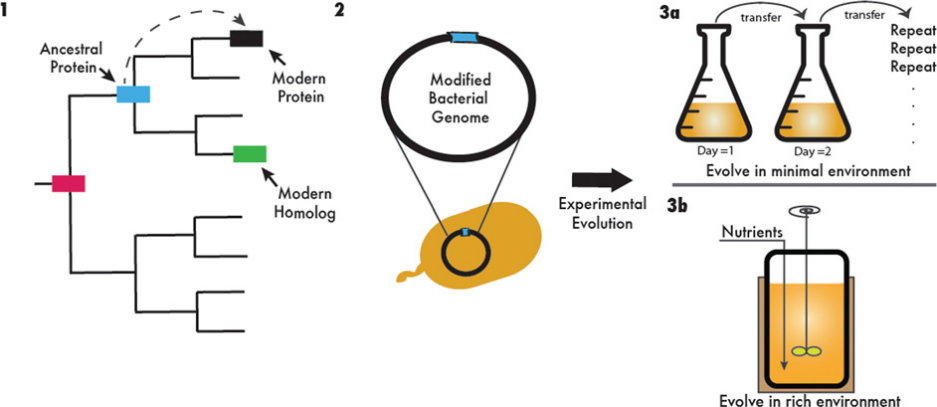
Pathways for network evolution Current advances in synthetic biology and experimental evolution allow us to reprogramme biological networks through integration of evolutionary history. One proposed application of synthetic biology, particularly ASR, should allow us to investigate how the molecular history of a given network component has influenced the evolution of the interaction network. Overall, the aim is to rewind and then replay the molecular tape of a protein's history in an extant organism in three steps. Step 1: using the tools from ASR, the evolutionary history of a modern protein (shown as a black box in the phylogeny tree) could be constructed through evolutionary algorithms. Inferred ancestral protein sequences (such as the blue and pink boxes) that share a direct line of evolutionary history with the extant protein could then be resurrected in the laboratory [66]. Replacing endogenous components of a modern network in a modern organism with the inferred ancestral genes would allow us to rewire modern systems within a historical context for comparative analysis. Alternatively, replacing endogenous components of a modern network with a modern protein homologue (green box) could be limiting, since a mutation occurred in the immediate history of this extant homologue could potentially prevent its interactions with nodes that have adapted to the other hub in a separate environment and thus exhibit non-swappable properties. Step 2: various methods exist to replace a modern gene with its resurrected counterpart in the exact chromosomal location [86]. Modifying an essential component of a protein network with an ancient protein will most likely cause the modern organism to be maladapted. This artificial gene component will trigger a strong selective pressure and thus will provide sufficient conditions for Darwinian evolution to take the lead and allow us to monitor evolution in action. Step 3: the experimental evolution approach is powerful, as it permits a high level of control, and a capacity to create and maintain a frozen fossil record of the evolved populations that may later be used for detailed analyses. Shown here are two alternative ways to set up an experimental evolution system in the laboratory. Bacterial populations can be propagated in a well-defined growth medium through daily serial dilution (Step 3a). Alternatively, populations could be continuously fed in a closed system, such as a chemostat under highly controlled environmental conditions (Step 3b). Conditions in which the evolutionary experiment is set up influence the selection pressure that the system experiences, and thus could potentially influence the evolutionary course to be taken by the population.

ASR of proteins infers the ancestral sequence of a common ancestor on the basis of a comparison of modern (or extant) sequences placed in an evolutionary context. The process has a level of confidence/certainty based on the models and algorithms used to infer ancestral states [67,68]. For over a decade, ASR has been making contributions to our understanding of evolutionary relationships by offering us an opportunity to determine the amino acid replacements responsible for changes in protein behaviour associated with adaptive events for particular molecular systems [69–72]. One proposed application of ASR would allow us to investigate how the molecular history of a given node has influenced the evolution of an interaction network. Specifically, by replacing endogenous nodes of a modern network and in a modern organism with the corresponding ancestral components, we can reprogramme modern networks within a historical context for comparative analyses (Figure 3, step 1). Therefore ASR could provide insights into the evolutionary past of proteins involved in interaction networks. Furthermore, applying laboratory evolution experiments into ASR, whereby the co-adaptation between the network containing ancient interaction partners and the whole organism is monitored, would provide insights into how these networks evolve and shape the evolutionary trajectory of a given organism. Such an approach could be further empowered by biochemical, structural and systems-level studies that allow us to identify the precise molecular location of mutations, but also the effects of such mutations at the organismal level.

## ANCIENT PROTEIN HUBS IN MODERN SYSTEMS

We have described recently such an approach for the EF-Tu (elongation factor Tu) protein in *Escherichia coli* [73]. In addition to serving an essential cellular function by carrying aminoacylated-tRNA complexes into the ribosome, EF-Tu is a hub protein of a very busy molecular interaction network, binding to a range of metabolic enzymes, chaperones, and structural and regulatory proteins [74–76] (Figure 2B).

EF-Tu proteins are ideal for evolutionary studies because they are highly adapted to their environments; thermostability profiles of EF-Tu proteins are linearly correlated with their individual host's optimal growth temperature [77]. As a result, EF-Tu proteins from mesophilic micro-organisms are not optimally functional at thermophilic temperatures and vice versa. Various *in vitro* studies support this observation. For example, we have shown that mesophilic EF-Tu can participate, although less efficiently than their thermophilic counterpart, in a cell-free translation system in which all other necessary components for translation besides EF-Tu are obtained from a thermophilic organism [78]. EF-Tu proteins also maintain their structural stability and primary function in divergent organisms through subtle adaptive mutations. For instance, EF-Tu from *Bacillus stearothermophilus* (with a growth temperature of ~60°C) and EF-Tu from *E. coli* (with a growth temperature of 37°C) share approximately 75% sequence identity (out of 400 residues) and share conserved structural features [79]. And yet, chimaeric forms of these two homologues, constructed by swapping functionally important domains between different thermophilic compared with mesophilic EF-Tu proteins, are shown to exhibit some level of *in vitro* catalytic activity [79,80]. In light of these results, one can ask, what kind of evolutionary mechanisms allow EF-Tu to respond to its environment so selectively while simultaneously maintaining overall protein and network structure, as well as organismal functionality?

One way to study how EF-Tu evolves within the context of the whole network would be to replace an endogenous EF-Tu with its evolutionary homologue within a host genome and then observe how the rest of the network and the cellular machinery adapt to the presence of this recombinant homologue. EF-Tu is suitable for such an investigation since the majority of the components in the EF-Tu interaction network are conserved in most micro-organisms, despite having evolved under distinct conditions and experiencing distinct external fluxes. Furthermore, current data suggest that when responding to environmental conditions, EF-Tu proteins retain their structural specificity and sustain their network's optimal global function. Such a high degree of structural and functional conservancy would suggest that a thermostable EF-Tu from thermophilic bacteria should exhibit some level of activity in mesophilic bacteria when inserted into the genome in place of the endogenous counterpart. In practice, however, the process is more complicated. The functionality of EF-Tu in the environment of a living cell may not be readily parallel with its *in vitro* activity measured in an observational environment such as a cell-free protein synthesis assay. As noted above, EF-Tu has many roles in the cell and interacts with multiple cellular partners that are involved in a variety of functions. As such, *in vitro* assays that fail to measure properties of a protein network obviously do not provide a complete understanding of a protein's function, with function being defined as how the protein contributes to the fitness of the host organism in a Darwinian context. Along these lines, two homologues may be experimentally interchangeable in an *in vitro* assay, but when one homologue is inserted into a different homologue's host, the host may not be viable because the foreign homologue cannot participate in the host's interaction network (Figure 3, step 1).

An alternative method to understand the evolution of protein networks would then be to reconstruct the ancestral gene/proteins of biological networks through ASR. Replacing the endogenous protein from a modern organism with an ancient form of the protein and then monitoring the real-time adaptation of the recombinant organism would allow us rewind the evolution of the protein and allow it to re-evolve in a modern context. Such an animation would be informative on the role history plays as a constraint shaping evolutionary trajectories. Predictably, modifying an essential component of a protein network, such as its hub, with an ancient construct is most likely to cause the modern organism to be maladapted when the ancient hub is not functionally equivalent to the modern hub. Such a strong selective pressure will likely cause the network having the maladapted hub to be the target of selection, allowing us to monitor the real-time adaptation of the protein network (Figure 3, steps 2 and 3).

A variety of ancestral EF-Tu proteins have been resurrected through ASR, and have been shown to demonstrate an increasing trend in their melting temperatures as one travels further back in evolutionary time, in agreement with the Earth's larger paleotemperature trend [81]. To examine the effects of disrupting the protein interaction network of EF-Tu, we replaced the modern EF-Tu in *E. coli* with a 500-million-year old evolutionary ancestor [73]. This ancestor's melting temperature is only a few degrees higher than *E. coli*'s EF-Tu (39.5°C) and the two proteins have high sequence identity (only 21 out of 394 amino acid differ). This similarity was sufficient to enable viability in the recombinant organisms, yet different enough to introduce a stress on the organism, as observed by a 2-fold increase in the bacterial doubling time [73]. The next step undoubtedly is to determine the underlying molecular mechanisms responsible for this increase in doubling time.

Swapping a modern hub or a modern node with its ancestral counterpart in the cell permits us to reprogramme a network within a historical context from which the mutational differences between the modern and ancestral proteins share a direct evolutionary connection. Such an approach will provide insights into evolutionary paths and constraints that shape the evolution of modern networks. A major challenge, however, would be to predict, *a priori*, the adaptive patterns taken by the ancient component as it adapts within a modern system. Our ability to predict adaptive patterns requires that we understand the selective constraints that act on the members of a protein network. It is reasonable to assume that proteins having numerous binding partners experience stronger selective constraints than other proteins having fewer binding partners, all other aspects being equal. Therefore it is reasonable to expect a network's hub to evolve more slowly than the nodes of the network [82,83]. As such, we may expect that evolutionary innovations proceed through mutations in the less-connected nodes of the network (e.g. as demonstrated by Jeong et al. [41], that highly connected proteins are more essential for survival than fewer connected proteins).

In total, there are various plausible scenarios for the adaptation of a recombinant network composed of both ancient and modern proteins. The network's nodes may respond to the maladapted hub through small adaptive steps thereby conserving the topology of the network. Alternatively, the ancient hub of the network may accumulate compensatory mutations that allow it to interact better with modern nodes. Furthermore, the functional incompatibilities of the historically chimaeric network may be compensated by the emergence of a different network (itself either new or currently present in nature, or possibly even ancient).

Considering the complexity of living systems, we are unlikely to predict the exact evolutionary trajectory traversed by a network containing an ancient component, but resurrecting ancient components of a protein network inherently implies practical trade-offs that must be considered during the design phase of any investigation. For instance, although replacing the modern component of a highly connected network with an ancient component is likely to create a strong selection pressure on the network, and thus allow us to monitor evolution of a network in action, it is also more likely to disable the entire network's function. Alternatively, replacing a less-connected node of a network with an ancient protein is like having a less deleterious effect, but this may also fail to narrow the selection pressure to network level. Finally, there are (perhaps unforeseen) limitations to the proposed approach regarding the extent to which we can be certain that a substituted protein in a modern organism actually recapitulates ancient evolutionary pathways.

## CONCLUSIONS

Understanding how biological systems operate requires a comprehensive grasp of how the components within such systems interact with one another and contribute to the organism's global function. In the present review we focused on the studies that view biological systems through the lens of protein interaction networks that ask, how do protein interaction networks evolve and give rise to functional biological systems? We also articulate on whether we can we set up experimental systems with tools drawn from molecular evolution, molecular and synthetic biology, and implement what we learn from evolution not only to understand how biology operates at a molecular level, but also to design synthetic biological systems with pre-programmed functions.

Our ability to synthetically design networks and complex systems that function the way we predict relies on our ability to design more stable network partners, and thus on our ability to decompose and reduce the systems into a few, yet optimal, number of interacting members. Attempts to experimentally dissect a cellular network, as we have discussed in the present review, vary and are far from simple. Current efforts to understand biological systems predominantly attempt to make the connection between organismal genotype and phenotype, and then mapping phenotype to fitness [84]. There is, however, a missing step in this flow chart; before connecting how genetic architecture gives rise to certain phenotypic outcomes, we need to understand how components that form the genetic make-up of a cell interact with one another, and this requires dissecting the biochemical and biophysical properties of cellular components and their interactions within the context of the whole system.

A reductionist, by training, will assume that the basic biochemical principles of individual members of a network (specifically, of an essential member of a network) will directly reflect on the network's global function, concluding that the sum of the individual behaviours of a network's components gives rise to the overall function. Biology operates far from reductionism, however. Cellular systems are elaborate; containing more abundant genetic make-up than seems necessary for the function where the sum of the individual members not always equal the whole. Therefore one way to study how networks function would be through evolving networks under certain constraints, define the redundant parts, and then to rewire the network lacking these parts.

Addressing this challenge to comparatively study biological networks at the systems level, we suggest in the present review a novel experimental approach that combines laboratory evolution experiments with ancestral sequence resurrection studies. We also suggest experimental evolution of synthetically altered networks in order to determine how systems-level changes occur in relation to network changes that are themselves responses to their micro and macro environments. Particularly, ASR studies could be used to identify the paleo-states of the modern proteins (be it a different physical or structural state, or a different pH tolerance level). Such protein level differences can then be used directly to determine how physical and historical constraints shape the system's evolutionary trajectory, if this protein also functions in an essential network of the cellular system.

A multidisciplinary approach to network evolution that combines systems biology with synthetic and evolutionary biology can provide a better understanding for how networks evolve. We anticipate that this combination will allow researchers to expand on what has evolved in nature by introducing or directing functionality into networks.
